# Respiration Based Non-Invasive Approach for Emotion Recognition Using Impulse Radio Ultra Wide Band Radar and Machine Learning

**DOI:** 10.3390/s21248336

**Published:** 2021-12-13

**Authors:** Hafeez Ur Rehman Siddiqui, Hina Fatima Shahzad, Adil Ali Saleem, Abdul Baqi Khan Khakwani, Furqan Rustam, Ernesto Lee, Imran Ashraf, Sandra Dudley

**Affiliations:** 1 Department of Computer Science, Khwaja Fareed University of Engineering and Information Technology, Rahim Yar Khan 64200, Pakistan; siddiqov@gmail.com (H.U.R.S.); hinafatimashahzad@gmail.com (H.F.S.); adilalisaleem@gmail.com (A.A.S.); furqan.rustam1@gmail.com (F.R.); 2 Management and Information Technology, Jubail Industrial College, Al Jubail 35718, Saudi Arabia; khan_ab@jic.edu.sa; 3 Department of Computer Science, Broward College, Broward County, FL 33301, USA; 4 Department of Information and Communication Engineering, Yeungnam University, Gyeongsan 38541, Korea; 5 School of Engineering, London South Bank University, London SE1 0AA, UK; dudleyms@lsbu.ac.uk

**Keywords:** machine learning, non-invasive emotion recognition, physiological signals, respiration rate, ultra-wide band

## Abstract

Emotion recognition gained increasingly prominent attraction from a multitude of fields recently due to their wide use in human-computer interaction interface, therapy, and advanced robotics, etc. Human speech, gestures, facial expressions, and physiological signals can be used to recognize different emotions. Despite the discriminating properties to recognize emotions, the first three methods have been regarded as ineffective as the probability of human’s voluntary and involuntary concealing the real emotions can not be ignored. Physiological signals, on the other hand, are capable of providing more objective, and reliable emotion recognition. Based on physiological signals, several methods have been introduced for emotion recognition, yet, predominantly such approaches are invasive involving the placement of on-body sensors. The efficacy and accuracy of these approaches are hindered by the sensor malfunctioning and erroneous data due to human limbs movement. This study presents a non-invasive approach where machine learning complements the impulse radio ultra-wideband (IR-UWB) signals for emotion recognition. First, the feasibility of using IR-UWB for emotion recognition is analyzed followed by determining the state of emotions into happiness, disgust, and fear. These emotions are triggered using carefully selected video clips to human subjects involving both males and females. The convincing evidence that different breathing patterns are linked with different emotions has been leveraged to discriminate between different emotions. Chest movement of thirty-five subjects is obtained using IR-UWB radar while watching the video clips in solitude. Extensive signal processing is applied to the obtained chest movement signals to estimate respiration rate per minute (RPM). The RPM estimated by the algorithm is validated by repeated measurements by a commercially available Pulse Oximeter. A dataset is maintained comprising gender, RPM, age, and associated emotions which are further used with several machine learning algorithms for automatic recognition of human emotions. Experiments reveal that IR-UWB possesses the potential to differentiate between different human emotions with a decent accuracy of 76% without placing any on-body sensors. Separate analysis for male and female participants reveals that males experience high arousal for happiness while females experience intense fear emotions. For disgust emotion, no large difference is found for male and female participants. To the best of the authors’ knowledge, this study presents the first non-invasive approach using the IR-UWB radar for emotion recognition.

## 1. Introduction

Emotions are an integral part of our everyday life that represent conscious and/or unconscious mental reactions to events, objects, and situations. Emotions are a combined form of feelings, thoughts, and behavior and show people’s psychophysiological reactions. Emotions affect the way people think, comply, and feel about people, things, and events. The most widely used emotion classification models are Ekman and pleasure, arousal, dominance (PAD) models [1]. The PAD emotional state model was developed by Albert Mehrabian and James A. Russell to define and measure emotional states. The PAD model describes continuous emotion in three dimensions of pleasure, arousal, and dominance [2,3,4]. Initially, it was used in an environmental psychology theory with the central assumption that environments influence individuals through their emotional effect [2]. PAD is a psychological model for estimating all the emotional states of humans with respect to pleasure, arousal, and dominance. Paul Ekman and his colleagues concluded that there are six basic emotions including disgust, fear, anger, happiness, sadness, and surprise [5], where each emotion has particular characteristics that allow them to be expressed to varying degrees [6]. Since emotions are basically genetically determined, distinct emotions are perceived similarly throughout most cultures or nations [7]. Many works employ dimensional representations to analyze human emotions in many emotional dimensions. Emotions are composed of five coordinated activities: mental situation assessment, clinical findings (central and autonomous nervous system response), actions, facial expressions, and thoughts [8]. The usage and role of emotion recognition are indispensable in a multitude of domains. For example, emotion detection plays an essential role in the field of medicine specifically for patients with psycho-neural disorder or patients with learning disabilities and autism. Children with autism spectrum disorder (ASD) typically find it difficult to understand, communicate and regulate feelings [9]. For advanced human-computer interface (HCI) designs, the interaction between robots and humans can be more realistic and dynamic if the emotional state of humans can be determined accurately. In this way, it can strengthen human-machine engagement by using emotional information during conversation.

The importance and diverse use of emotions in artificial intelligence, psychology, cognitive neuroscience, and advanced robotics, etc., has made emotions recognition extremely significant. Consequently, several methods have been developed for emotion recognition over the past years. Predominantly, such methods are based on individual physical signs that include different expressions, speech, body movement, and gestures, etc. [10]. So, facial expressions, speech, behavior, and physiological signals can be used for emotion recognition [10,11,12]. Depending upon the nature of the data used for emotion recognition, these methods fall under subjective and objective categories. The first three methods are subjective as it is easier for people to hide their genuine emotions by deliberately changing their voice, manipulating their facial expression, and altering their behavior [13]. Physiological signals-based methods are objective and more reliable where the signals generated by central nervous systems such as electroencephalogram (EEG) are used for emotion recognition. Objective methods are less susceptible to manipulation and show better performance [14]. Thus, emotion recognition mapping with physiological signals intuitively seems more reasonable. Several methods record physiological signals such as Galvanic skin response (GSR), EEG, Electrocardiogram (ECG), and Electromyography (EMG) for the electric activity of the heart, skin, muscles, and brain, respectively. Understanding emotional response using the physiological signals is promising because they show unconscious representations and are not consciously manipulated by humans [15].

Emotion recognition methods using physiological signals involve invasive technologies or on-body sensors for signals measurement which make them prone to error. For example, the electroencephalogram cap used in EEG is comprised of electrodes, amplifiers, and analog to digital converters for recording human brain activity and it is to be placed on the human head for this purpose [16]. EEG signals analysis is challenging due to being non-stationary and the influence of complex environmental factors. The EEG signals, for example, are noisy and highly susceptible to environmental interference due to their low amplitude (i.e., 50 μV to 100 μV) [14]. Similarly, GSR records skin conductance that occurs due to sweat gland activity and involves skin electrodes mounted on hand and foot regions. Research shows that the data and the accuracy of GSR-based methods are sensitive to several factors such as inappropriately worn devices, unrestricted movements of participants and gender, etc. [17,18]. Additionally, many humans do not feel comfortable with the on-body sensors, and their movements introduce noise and error in the collection that affects the performance of emotion recognition [19]. Keeping in view the challenges and limitations associated with invasive emotion recognition approaches, a non-invasive emotion recognition method is a compelling necessity. This study utilizes impulse radio ultra-wideband (IR-UWB) to propose a non-invasive emotion recognition method and makes the following contributions:

- A non-invasive emotion recognition method is proposed using IR-UWB radar. Emotion recognition is carried by measuring the chest movement of the subjects without involving on-body sensors and invasive technology.
- A dataset of IR-UWB data is maintained involving 35 participants in total, including both males and females.
- An approach is presented to measure respiration per minute (RPM) from the measured chest to IR-UWB distance data. Results of the proposed approach are verified by a commercial pulse oximeter.
- For identifying the emotions, the IR-UWB data is complemented with the machine learning approach where ensemble voting is utilized including both hard voting and soft voting.
- Analysis has been carried out for male and female participants separately to present the differences with respect to gender. The emotion recognition performance of the ensemble models is compared with other machine learning models.

The rest of the paper is structured as follows. Section 3 discusses important research works related to the current study. The process of data collection using IR-UWB proposed research methodology, and its related contents are described in Section 4. Section 5 provides the analysis and discussion of results. In the end, the conclusion is given in Section 6.

## 2. Background on Suitability of Respiration and IR-UWB for Emotion Recognition

### 2.1. Respiration Patterns during Emotions

Physiological signals record the response of various human organs such as the brain, heart, and sweat glands, etc. when facing situations involving fear, anger, love, and hatred, etc. For recording physiological signals, several devices are used; for example, EEG for brain activity, GSR for skin conductance and photoplethysmogram (PPG) for measuring the volume of blood flow [18]. Research shows patterns from such and similar other physiological signals can be subsequently used for human emotion recognition [20].

Of the physiological signals recorded during emotion arousal, respiration is more apparent and prevalent. The respiration patterns show a high correlation with human emotions, e.g., fast breathing may be caused excitement due to happiness, anger, or anxiety [21]. Happiness and other positive emotions have a substantial impact on respiratory changes [22,23]. The high frequency of heart rate variability is substantially influenced by respiration due to heart rate increase during inspiration and heart rate decrease during expiration, a phenomenon called respiratory sinus arrhythmia (RSA) [24,25]. Happiness has a variable effect on breathing rate depending on how arousal one is, whilst arousing one increases the respiration rate [26]. Similarly, humans tend to suppress their breaths when having emotions of disgust [22,23]. Research also shows [22,23,27] that humans demonstrate shallower and faster breathing when facing fear. In the light of these findings, this study adopts the respiration rate measurement for predicting various emotions using the IR UWB radar.

### 2.2. Suitability of IR-UWB for Emotion Recognition

Currently, different devices are deployed for physiological signals measurement. For example, EEG is used for recording the electrical activity of the human brain when facing certain emotions [28]. The heart rate is monitored using electrocardiography (ECG) involving the famous 12-lead ECG technique where nine sensors are placed on arms, legs, and the chest. Heart signals show different patterns during different emotions. GSR is used to record continuous skin conductance of the human skin. GSR shows the changes in sweat reaction caused by the change in the sympathetic nervous system [29]. Similarly, skin temperature measurement, electromyogram (EMG), and electrooculography (EOG), etc. are among the commonly used devices for recording physiological signals. For respiration rate monitoring, the use of radar has been increased over the past few years. Consequently, several types of radars have been leveraged for recording respiration patterns such as continuous wave Doppler radar, ultra-wideband radar, frequency modulated continuous wave (FMCW) radar and stepped-frequency continuous wave radar [30]. Radar can provide larger detection areas as compared to other approaches, for example, video and thermal cameras, and can be used to monitor multiple subjects. Provided the proper body position of the subject, higher accuracy of up to 97% is achievable for respiration rate monitoring using FMCW radar [31]. Radar shows high accuracy for monitoring the tiny movements of the chest wall and can provide accurate respiration-related movements.

## 3. Related Work

On account of large interest in emotion recognition during the past few years and the importance of respiration rate association with emotions, several studies have presented methods and systems to recognize emotions based on respiration rate.

Augsburg’s dataset of physiological signals has been used for the classification of emotions by [32]. Augsburg dataset consists of twenty-five records for four emotions including joy, sadness, pleasure, and anger where these emotions were triggered by the musical induction process. Four forms of physiological signals have been obtained during this process: ECG, EMG, respiration, and skin conductivity (SC). The ensemble empirical mode decomposition (EEMD) approach is used to extract time-domain non-linear, time-frequency, and intrinsic mode (IMF) features. The C4.5 decision tree (DT) is used to limit the number of features to five optimal features with a major contribution to classification. The correct classification rate (CRR) is used to measure the output and results show a CRR of 88% using the selected features.

The study [33] uses a dataset for emotion analysis using physiological signals (DEAP) dataset containing ECG, GSR, blood pressure, breathing, skin temperature (ST), EMG, and Electrooculogram signals of thirty-two participants (16 of each gender) with ages from 19 to 37 years. Data are recorded when the subjects are watching forty-one-minute music video clips with a rating of 1-9 which is negative/low to positive/high using arousal, valence, and liking. The study uses only two ECG and respiration signals for emotion recognition. Thirteen features are obtained at a sampling rate of 512 Hz. Respiration rate (RR) interval, low frequency (LF), heart rate (HR), and high frequency (HF), RSA power, RSA frequency, and RSA amplitude are among the thirteen cardiac features. Breathing frequency and amplitude, RSA amplitude ratio to respiratory oscillation, respiratory and RSA frequency difference, the phase difference of respiration and RSA, the slope of phase difference, and standard deviation are calculated. For emotion classification, an SVM classifier with a multilayer perceptron kernel is used. Low/high liking, positive/negative valence, and low/high arousal are performed for the classification of ECG and respiration signals. Using the ECG signals, accuracy scores of 74%, 71%, and 72% are obtained for liking, arousal, and valence, respectively. Respiration rate shows an accuracy of 73% for liking, 72% for arousal, and 70% for valence. On the other hand, with a combination of HR and RR, the classification accuracy is increased to 76%, 74%, and 74% for liking, arousal, and valence, respectively.

Along the same lines, ref. [34] uses various physiological signals such as ECG, ST, GSR, EMG, HR, respiration rate, blood oxygen level, systolic blood pressure (SBP), diastolic blood pressure (DBP), and blood volume pulse (BVP) to distinguish anger, pleasure and neutral emotions. A total of three stable male subjects aged 18 to 19 years participated in the data collection process. Zephyr BioPatch chest brace is used for the collection of ECG and respiration signals while E4 wrist-band is used to capture GSR, ST, and (BVP). The SBP and DBP are calculated using CONTEC which is an off-the-shelf Bluetooth-enabled blood pressure device. Blood oxygen level is measured by a pulse oximeter. The Zephyr chest strap is worn under the pectoral muscle for data collection. Blood glucose readings are taken twice at the beginning and the end of the trial. Baseline signals are captured while the participant is sitting comfortably, followed by inducing the joy emotion joy using video clips. For inducing anger, cognitive techniques are applied. The physiological signals of each subject are recorded twice in the state of the induced emotion. Four physiological signs SBP, DBP, EMG, and blood oxygen levels are omitted because they do not contribute much to the classification of joy and anger emotions. Bandpass filtering is used for ECG, respiration, GSR, ST, HR, and BVP signals. Because the dataset is limited (two instances for each emotion class), predictive and statistical analysis of the data is carried out. The emotion is classified as happiness when the signal has high GSR, HR, and moderate respiration value while anger has low GSR, ST, high respiration, and moderate HR values.

ECG, RR, blood pressure, and respiration inhalation, and exhalation temperature are used for emotion classification in [35]. Several sensors are used to capture four physiological signals from a single subject at the time of the induction of emotion through a three-minute video clip. The movie clip is played at a one-meter distance at the laptop and the signals are recorded for one minute. By applying the Low-Pass filter to the raw ECG and respiratory signal, the noise is reduced. Afterward, nineteen statistical, temporal, and spectral features are extracted for emotion recognition. An artificial neural network (ANN) with two hundred hidden layers is used for classification. A total of six emotions happy, sad, fear, disgust, anger, and surprise are classified with an average accuracy of 80%.

In [36] MAHNOB-HCI physiological signal dataset is used for the classification of emotions in the arousal valence model. ECG, respiration, skin temperature, and galvanic skin reaction are used for emotion classification. The MAHNOB-HCI multimodal dataset contains data for twenty-four subjects using twenty video samples. The signal of the first and last thirty seconds is omitted because of neutral emotion. Butterworth filter is applied on GSR, ECG, and respiration signal with a cutoff frequency of 0.3 Hz, 0.7 Hz, and 1 Hz, respectively. Heart rate variability (HRV) is calculated from the ECG signal and respiration rate from respiration amplitude. A total of one hundred sixty-nine features are extracted from these signals. An SVM with different kernels is used for classifying the samples into high and low arousal, negative and positive in valence. SVM with RBF kernel shows a better accuracy of 68.5% for arousal and 68.75% for valence class.

A physiological signal interpretation framework, Emo-CSI, is presented for emotional classification in [37] which uses heart rate, respiration pattern, skin humidity, and strength to recognize emotions of pleasure, displeasure, calm, neutral, and excited emotions. Twenty-three subjects with ages from 20 to 27 are included in the data collection process seconds comprising ten males and thirteen females. Emotions are induced using pictures and matching sounds. A total of thirty-two features including average, maximum, minimum, and standard deviation, etc. are extracted from physiological signals to support prediction using SVM, DT, and artificial neural network (ANN). SVM outperforms the other two classification models with an accuracy of 55.45% and 59% for valence and arousal classes, respectively.

Respiration data are used in [38] where the emotions are classified using that are Fast Fourier Transform (FFT) and machine learning models separately. Twenty-five males and females with ages from 18 to 25 participated in the experiments where seven movie clips are shown to subjects for six emotions inducing happiness, sadness, surprise, anger, anxiety, and disgust. The study uses the BIOPAC instrument, airflow sensor, and a mouthpiece for collecting respiration, and breathing patterns. FFT-based classification achieves an 80% accuracy while LR obtains an 80% recall. A system using HRV signal based on respiration rate for emotion classification is proposed in [39]. Twenty-five subjects (12 males & 13 females) with ages from 18 to 35 are involved in experiments for anger, fear, joy, and sadness emotions. ECG and respiration signals are recorded using a BIOPAC device. The largest peak from the bandpass filtered respiratory signal is used to calculate respiratory frequency. The area under the receiver operating characteristic curve (AUC) is calculated to find the capability of features in emotion classification. Features having AUC greater than or equal to 0.70 are considered for further process. Accuracy of two-class classification (relax vs. joy, joy vs. sad, and joy vs. anger) is 79.2%, 77.8%, and 77.3%, respectively.

The literature study has several important findings. First, despite the higher classification accuracy, predominantly, the used methods involve invasive or on-body sensors. Invasive sensors can be used in virtual or controlled environments but for real-life situations their practicability is limited. Often causing attention or inconvenience, the data collection process becomes erroneous. Secondly, respiration rate has been employed largely in recent studies and is a potential candidate for accurate emotion detection. Thirdly, UWB radar has not been studied extensively and requires further research efforts to explore its full potential for emotion recognition. Keeping in view these points, this study presents a non-invasive emotion recognition approach by employing IR-UWB for recording physiological signals.

## 4. Materials and Methods

### 4.1. Impulse Radio-Ultra Wide Band Radar

Time domain and extreme spectrum commercialized IR-UWB radar appeared in the late 1990s [40,41]. It is a developing technology that was originally used by the United States (US) army in the 1970s. The Federal Communications Commission (FCC) of the US has allocated a bandwidth of 7.5 GHz for UWB signals. A signal is called UWB if its bandwidth exceeds 500 MHz and its frequency range falls in the ranges from 3.1 GHz to 10.6 GHz [40]. Despite the high data rates, UWB signals have a limited transmission capacity. IR-UWB radar creates high bandwidth signals by transmitting very short-duration pulses in the range of nanoseconds. The IR-UWB radar has no privacy concerns and is unaffected by environmental variables. Because of the IR-UWB radar’s extremely low emission power, it has no adverse effect on the human body and passes the safety standards. Because of its non-ionizing, non-intrusive, non-tackling, no blind spot angles, and ability to penetrate a range of materials or barriers, the IR-UWB radar offers benefits over other existing tools for recording the physiological signals [42,43,44].

### 4.2. Proposed Research Methodology

**Figure 1:**
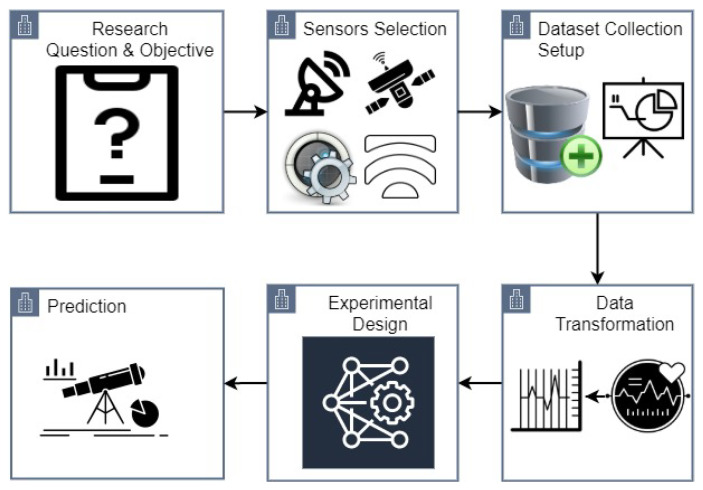
Steps carried out in the research.

Figure 1 shows the architecture of the research methodology followed in the current study. Initially, the objectives of the study are defined including the emotion prediction, types of emotions, and the non-invasive prediction procedure. The second step is the selection of appropriate sensors for which a sensor is to be decided regarding its ease of use, cost, robustness, and ease of deployment for practical applications. It is followed by the data collection set up including the decisions regarding the number, age, and gender of the participants and the data collection procedure such as static or dynamic. Data cleaning and transformation procedures are defined to remove the noise and increase data quality. Then the experimental design is made with the selection of appropriate tools to be used for data collection routines and sensors placement for experiments, etc. In the end, prediction is carried out using the selected machine learning and deep learning models which are selected based on their reported results and suitability with respect to the task at hand.

#### 4.2.1. Research Questions and Objectives

In the first phase of this research, objectives are defined. The primary objective of this research is to devise a non-invasive method for detecting human emotions. Initially, three emotions are considered to validate the feasibility of non-invasive emotion detection. In addition, the study also aims to obtain higher accuracy using single sensor data which means that data preparation and transformation should also be studied.

#### 4.2.2. Sensors Selection

Appropriate data is the fundamental element for obtaining accurate human emotions and data quality is directly linked with the data collection sensor. Besides data quality, other important aspects are the cost, availability, and ease of deployment of the data collection sensor. Currently, GSR, ECG, EEG, EMG, respiration, and SC physiological signals are used for emotion detection, each with its own merits and demerits. Keeping in view the results reported from different research studies [35,38,39], respiration rate has been selected for emotion detection. IR UWB radar’s tolerance to environmental noise, human eye safety, reduced cost, and ease of deployment are convincing traits for using it for respiration data gathering.

#### 4.2.3. Data Collection Setup

**Figure 2:**
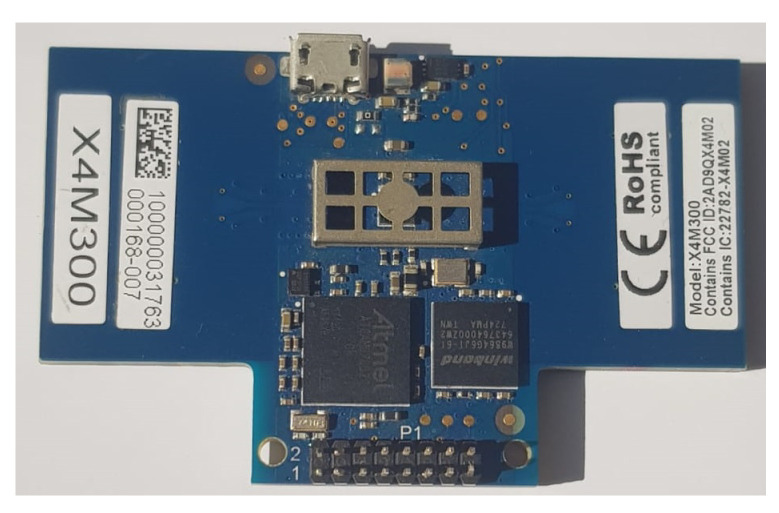
The X4m300 IR-UWB radar used for data collection.

For collecting the physiological signals, this study uses an X4m300 IR-UWB radar, as shown in Figure 2. It has a configurable frame size with a detection time of 1.5 to 3.5 s while the detection zone is programmable to up to 9.4 m [45,46].

The IR-UWB radar runs on the X4 chip framework (system on chip (SoC)) with unlicensed core frequencies of 7.29 GHz or 8.748 GHz at 1.4/1.5 GHz (−10 dB) bandwidth. It is operated by a very sensitive XeThru X4 UWB chip that senses smaller motion and has the best signal-to-noise ratio. It has built-in antennas and has a very high-resolution rate due to short pulse transmission at nanoseconds [47]. The built-in firmware creates a baseband signal that covers 9.4 m beginning at 0.18 m when the board’s default settings are used. This distance is split into 181 bins with 0.0514 m bin lengths. For the current study, the effective range is between 0.2 to 1.6 m. A baseband data frame counter is given with a frame size of 0.2 to 1.6 m. The corresponding bins for this effective range start with the 2nd bin at 0.282 m and end at the 28th bin at 1.569 m. A baseband data frame counter is given with a frame size of 2${}^{32}$. For each radar frame, i.e., the X4 UWB radar SoC output, the frame counter is increased by one. The frame counter does not reset when a profile is stopped or started. When the limit is hit, the frame counter resets to 0. The frame counter will be reset if the X4 UWB radar SoC is reset or the sensor module’s power is toggled [48].

**Figure 3:**
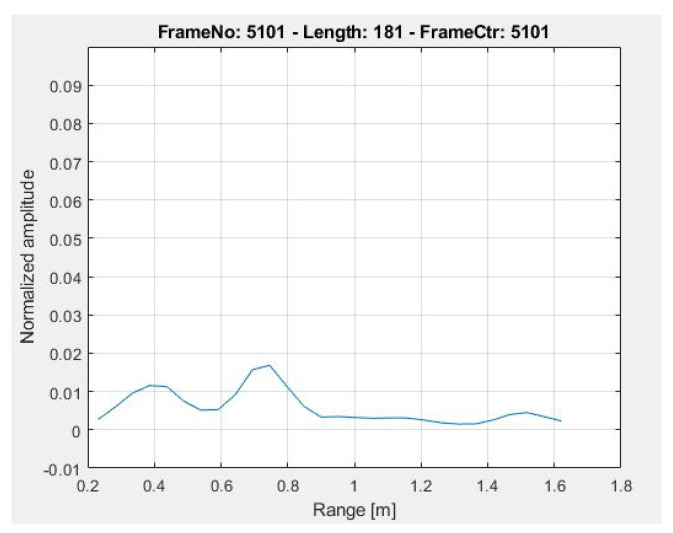
UWB radar signals during chest movement.

The IR-UWB radar is used to record the respiration process where the chest moves due to the lungs’ respiratory operations. During the respiration process, the inhalation fills the human lungs with oxygen and expands the upper portion of the body and the space between the radar and the chest of the human body reduces, whereas the exhalation does exactly the opposite. A plot of the resulting distance is given in Figure 3 where the impact of inhaling and exhaling is shown. The inhalation increases the distance of the chest from the radar while the exhaling decreases this distance. To count the number of expands and contracts of the chest due to respiration the area under the curve is considered.

For data collection, the frequency of radar is set to 20 Hz. Subsequently, 1200 (20 × 60) areas under the curve are estimated in one minute. The data are recorded for 5 min and contain 6000 (20 × 60 × 5) area values under the curve. A total of 35 subjects participated in the data collection process including 14 males and 21 female participants of age ranges between 18 to 30 years and the average age of 24 years. The subjects are wearing seasonal clothes during the experiment and no special clothes are worn for the data collection. Research shows that the UWB radar is not influenced by the clothes and if slight variations are caused, they can be moved during the data preprocessing using the low pass filter [49,50]. An ethical approval statement is designed and approved by the Khwaja Fareed University of Engineering and Information Technology (KFUEIT) ethical committee and a consent form is signed by each subject. The testbed is set up in the Computer Science Department lab in KFUEIT. The nature and process of the whole experiment are explained and demonstrated to the subjects before data collection.

**Figure 4:**
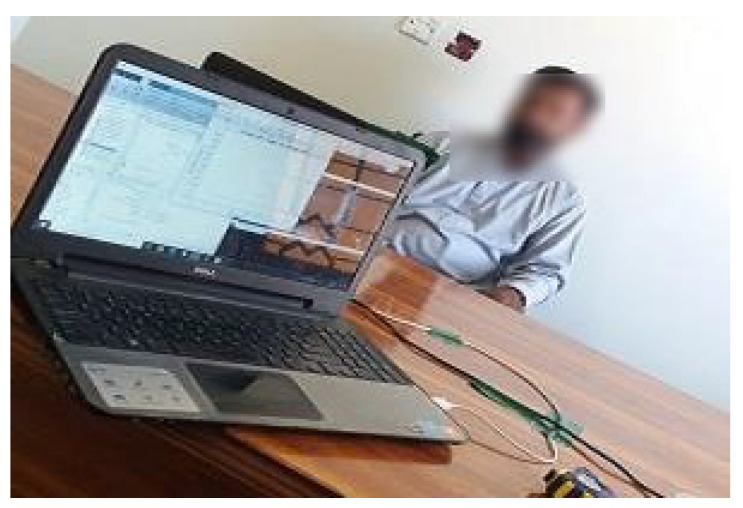
Subject sitting in front of radar while watching videos.

A total of nine videos have been selected to induce emotions and three emotions are considered for this study including happiness, fear, and disgust. Each video lasts for five minutes. Happiness is triggered by showing comedy movie clips while fear by showing clips of horror movies. Disgust emotion is induced by showing movie clips of persons eating distasteful food. Subjects are asked to enter the room alone and sit on the chair facing the IR-UWB radar. A distance of one meter from the IR-UWB is maintained for the person. This distance is selected based on the best view of the video clips from the sitting place. Figure 4 shows the experiment setup for data collection.

#### 4.2.4. Data Transformation

**Figure 5:**
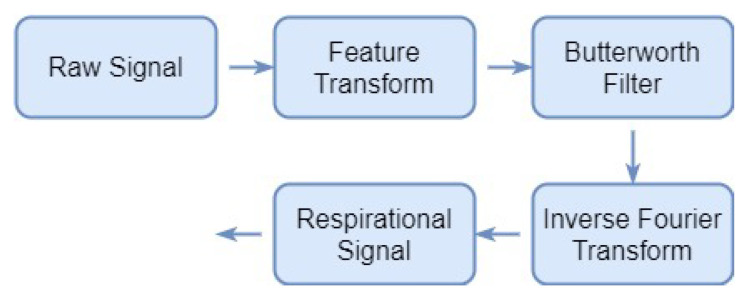
Steps involved in data cleaning and acquiring respiration data.

The collected data for area values under the curve comprises respiration, pulse, and noise including heartbeat, belly movement, eye-blinking, eyeball movement, and other ambient motions. However, the proposed method requires only the respiration patterns for which respiration signals are obtained from the collected data. Steps involved in data cleaning and obtaining the respiration data are portrayed in Figure 5.

Using the Fourier transform, the frequency spectrum of the gathered signal is obtained. The maximum frequency of an adult’s respiration rate is 0.4 Hz [44,51,52,53,54]. To get the respiration signal, a filter with a cut-off frequency of 0.4 Hz is required. For the normalized frequency in this study, the cut-off frequency of 0.4 Hz shifts to 0.04. Thus, a respiration signal is extracted by applying a tenth-order low-pass Butterworth filter with a cut-off frequency of 0.04 to remove the higher frequency noise. The Butterworth filter is a digital filter with a very smooth passband frequency response curve. The square amplitude response function of the filter is

(1) $${\left|H\left(jw\right)\right|}^{2}=\frac{1}{1+{\left(\frac{w}{w_{C}}\right)}^{2N}}$$

where *N* denotes the filter’s order, which is a positive integer, and $W_{c}$ is the low-pass filter’s cutoff frequency. In this study, N=10 and $W_{c}=0.04$.

#### 4.2.5. Experimental Design

For emotion recognition, this study uses the respiration signals acquired from IR-UWB radar. However, the raw signals are not used for emotion classification. Instead, respiration per minute (RPM) is leveraged to this end. For obtaining the RPM, initially, the data from IR-UWB radar are collected for the inhaling and exhaling process.

**Approximation of area under the curve** is performed using the Trapezoidal rule. For this purpose, the area under the curve for each frame is found. The trapezoidal rule evaluates the area under the curve by splitting the area into trapezoids, unlike the Reimann sums which follow a rectangular approach [55]. Let f(x) be continuous signal on [a,b], the interval [a,b] can be partitioned into *n* equal subintervals where the width of each is

(2) $$\Delta_{x}=\frac{b-a}{n},\;a=x_{0}<x_{1}<x_{2}...<x_{n}=b$$

Trapezoidal rule to approximate $\int_{a}^{b}f\left(x\right)dx$ is given as

(3) $$\int_{a}^{b}f\left(x\right)dx\approx T_{n}=\frac{\Delta_{x}}{2}\left[f\left(x_{0}\right)+2f\left(x_{x}\right)+...+2f\left(x_{n-1}\right)+f\left(x_{n}\right)\right],$$

where $\Delta_{x}$ is given in Equation (2) and $x_{i}=a+i\Delta_{x}$.

The Trapezoidal rule on 60,000 frames, provides 60,000 area values of the curve present in each frame. These values correspond to lungs inhaling and exhaling, the area under the curve increases during the inhaling process and vice versa. Subsequently, Fast Fourier Transform (FFT) is applied to the values obtained from the Trapezoidal rule. FFT can turn a time-domain signal f(t) into a frequency domain signal F(jw). The Formulae (4) and (5) are for Discrete Fourier Transform (DFT) and the inverse transform.

(4) $$X\left(k\right)=\sum_{n=0}^{N-1}x\left(n\right)e^{-jk(\frac{2\pi }{N})},\;k=0,1,2,3,...,N-1$$

The discrete Fourier transform converts the time-domain sequence x(n) into the discrete frequency domain signal X(k). The Inverse Discrete Fourier Transform (IDFT) formula is as follows

(5) $$x\left(n\right)=\frac{1}{N}\sum_{n=0}^{N-1}X\left(k\right)e^{jk(\frac{2\pi }{N})n},\;k=0,1,2,3,...,N-1$$

FFT is an enhanced form of DFT that greatly speeds up the calculation time of DFT. The FFT is used in the proposed work for the curve x(n)

(6) x(n),n=0,1,2,...,N−1

**Obtaining RPM from IR-UWB data** involves processing the data through several steps. The trapezoidal rule applied data is transformed into the frequency domain using FFT. Subsequently, the Butterworth filter is applied to extract the data related to respiration only. The Butterworth filtered data is used to find the peaks which represent the inhale process. For peaks, high movement locations in the data are to be found. Each round of inhaling and exhaling is regarded as one respiration. Finally, RPM can be obtained using

(7) $$RPM=\frac{n_{p}}{T}$$

where $n_{p}$ refers to peaks (data magnitude ≥α) while *T* shows the time in minutes.

For emotion recognition, machine learning algorithms are trained and tested using the obtained RPM information for each emotion.

#### 4.2.6. Prediction

Several supervised machine learning models are applied for emotion recognition on IR-UWB obtained data. These models are selected based on the results reported in other research works and include K nearest neighbor (KNN), extra tree classifier (ETC), AdaBoost classifier (ADB), gradient boosting machine (GBM). To obtain high classification accuracy, several hyperparameters have been fine-tuned for these models and a complete list is provided in Table 1.

**Figure 6:**
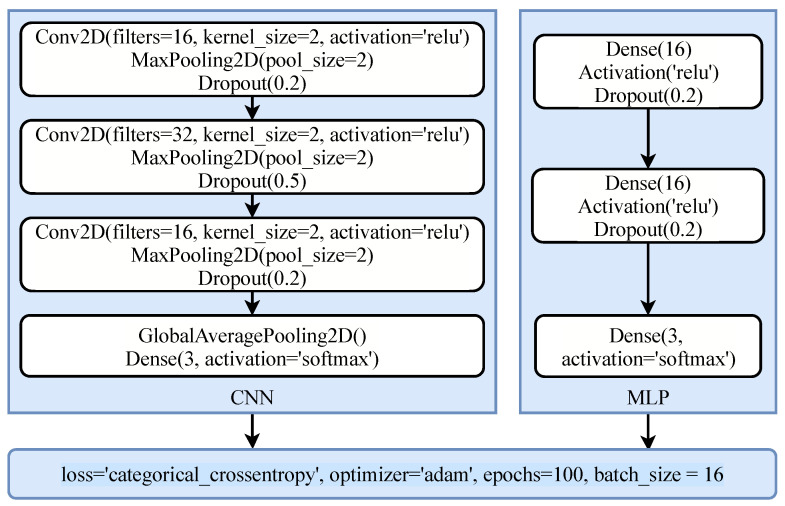
Architecture of the deep learning models used for experiments.

Besides using the machine learning models, two deep learning models have been employed as well for emotion recognition problems. For this purpose, multi-layered perceptron (MLP) [56] and convolutional neural network (CNN) [57] have been used so that a performance comparison can be made between the machine and deep learning approaches for the task at hand. The architecture of the models is customized in terms of the number of layers and neurons, and several other parameters such as optimization model, learning rates, and activation functions. A complete list of models’ parameters and architecture is provided in Figure 6. Each model is compiled with ‘categorical_crossentropy’ loss because of multi-class data and ‘adam’ optimizer is used for optimization. Deep learning models are fitted with a batch size of 16 and 100 epochs are used to train the models. For avoiding the overfitting problem, dropout layers with different rates are used both in CNN and MLP networks.

### 4.3. Proposed Hard Voting and Soft Voting Models

**Figure 7:**
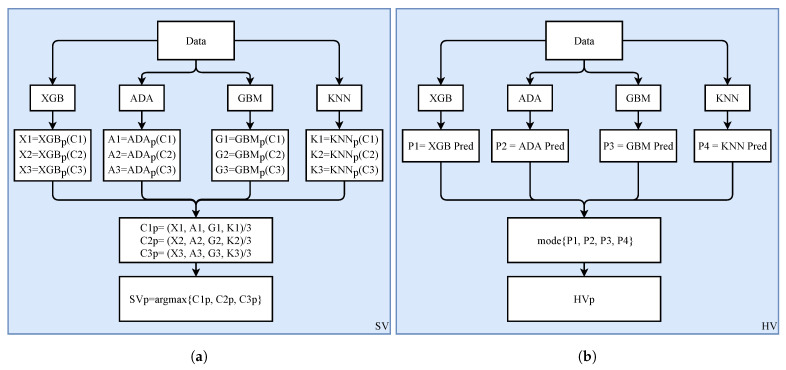
The architecture of ensemble models, (**a**) Soft voting, and (**b**) Hard voting.

Besides the machine learning models, this study proposes two ensembles of XGB, ADB, GBM, and KNN which use hard voting (HV) and soft voting (SV) criteria to make the final prediction. The architectures of both models are shown in Figure 7. SV works based on probability of each class as predicted by each model and these probabilities pass through average criteria. In the end, the argmax function is used to average the probabilities to compute the final prediction [58]. In SV model, $X_{1}$, $X_{2}$, and X−3 are the probabilities by XGB for class 1 ($C_{1}$), class 2 ($C_{2}$), and class 3 ($C_{3}$). For HV model, $P_{1}$, $P_{2}$, $P_{3}$, and $P_{4}$ are the predictions by each model and class with more votes will be final class [59]. The $C1_{p}$, $C2_{p}$, and $C3_{p}$ are the average probabilities for C1, C2, and C3. $SV_{p}$ is the final prediction by using soft voting criteria and $HV_{p}$ is the final prediction using hard voting criteria.

Algorithm 1 shows the steps followed by the ensemble model based on the hard voting criteria. The TXGB, TADA, TGBM, and TKNN represent the trained XGB, ADA, GBM, and KNN models on the feature vector of different emotions. Each model predicts a given sample with respect to one of three emotions. The prediction from each of these models has one vote and the final prediction $HV_{Pred}$ is based on the majority of the given models’ prediction for a particular emotion.
**Algorithm 1** HV Algorithm for Emotion Prediction**Input:** Emotion RPM**Output:** Happiness, Fear, or Disgust 1: TXGB←TrainedXGB 2: TADA←TrainedADA 3: TGBM←TrainedGBM 4: TKNN←TrainedKNN 5: **for** *i* in Dataset **do** 6:    $XGB_{Prediction}\leftarrow TXGB\left(i\right)$ 7:    $ADA_{Prediction}\leftarrow TADA\left(i\right)$ 8:    $GBM_{Prediction}\leftarrow TGBM\left(i\right)$ 9:    $KNN_{Prediction}\leftarrow TKNN\left(i\right)$10:    $HV_{Pred}\leftarrow argmax\left\{XGB_{Prediction},ADA_{Prediction},GBM_{Prediction},KNN_{Prediction}\right\}$11: **end for**12: Output:Happiness|Fear|Disgust←HVPrediction

Algorithm 2 shows the working steps of the decision made under the soft voting criteria. Similar to the hard voting scheme, TXGB, TADA, TGBM, and TKNN are the trained XGB, ADA, GBM, and KNN models. A probability score of each emotion is predicted by each trained model. For example, $HappyPob_{XGB}$ represents the probability score for happy emotion from the trained XGB model. Similarly, XGB provides the probability score for disgust and fear emotions. For making the final prediction, $Happy_{Prob}$, the average probability score for happy emotion, is calculated from each trained model and the highest probability score gives the final prediction $SV_{Pred}$.
**Algorithm 2** SV Algorithm for Emotion Prediction**Input:** Emotion RPM**Output:** Happiness, Fear, or Disgust 1: TXGB←TrainedXGB 2: TADA←TrainedADA 3: TGBM←TrainedGBM 4: TKNN←TrainedKNN 5: **for** *i* in Dataset **do** 6:     $HappyPob_{XGB}\leftarrow TXGB\left(i\right)$ 7:     $FearPob_{XGB}\leftarrow TXGB\left(i\right)$ 8:     $DisgustPob_{XGB}\leftarrow TXGB\left(i\right)$ 9:     $HappyPob_{ADA}\leftarrow TADA\left(i\right)$10:    $FearPob_{ADA}\leftarrow TADA\left(i\right)$11:    $DisgustPob_{ADA}\leftarrow TADA\left(i\right)$12:    $HappyPob_{GBM}\leftarrow TGBM\left(i\right)$13:    $FearPob_{GBM}\leftarrow TGBM\left(i\right)$14:    $DisgustPob_{GBM}\leftarrow TGBM\left(i\right)$15:    $HappyPob_{KNN}\leftarrow TKNN\left(i\right)$16:    $FearPob_{KNN}\leftarrow TKNN\left(i\right)$17:    $DisgustPob_{KNN}\leftarrow TKNN\left(i\right)$18:    $Happy_{Prob}\leftarrow \left(HapypPob_{XGB}+HappyPob_{ADA}+HappyPob_{GBM}+HappyPob_{KNN}\right)/4$19:    $Fear_{Prob}\leftarrow \left(FearPob_{XGB}+FearPob_{ADA}+FearPob_{GBM}+FearPob_{KNN}\right)/4$20:    $Disgust_{Prob}\leftarrow \left(DisgustPob_{XGB}+DisgustPob_{ADA}+DisgustPob_{GBM}+DisgustPob_{KNN}\right)/4$21:    $SV_{Pred}\leftarrow argmax\left\{Happy_{Prob},Fear_{Prob},Disgust_{Prob}\right\}$22: **end for**23: Output:Happiness|Fear|Disgust←SVPrediction

## 5. Results and Discussion

**Figure 8:**
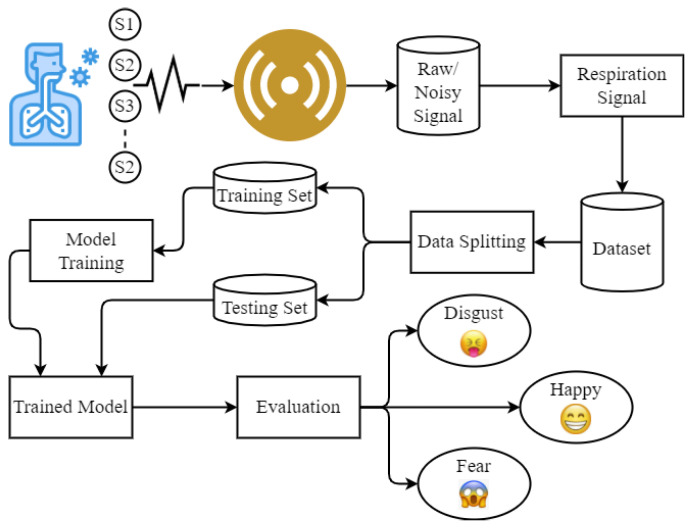
Architecture of the proposed methodology.

Figure 8 shows the steps followed to perform experiments. It involves data collection, data cleaning to obtain the respiration signal, followed by RPM gathering, data split, and training and testing.

### 5.1. Results of RPM Using Machine Learning Models

**Figure 9:**
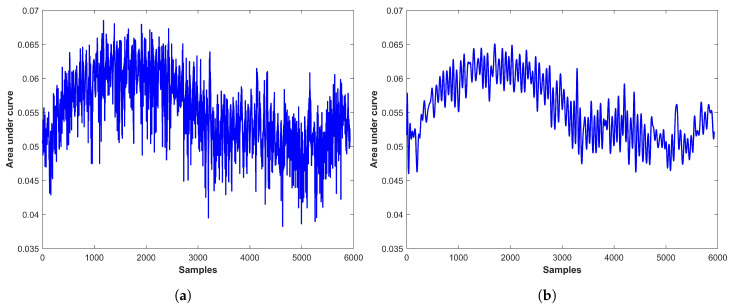
Data processed for RPM estimation, (**a**) Noisy data, and (**b**) Clean data.

**Figure 10:**
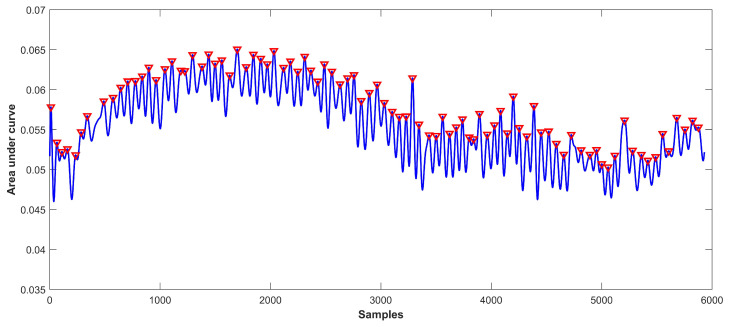
Detected peaks from the cleaned data.

Since the training and prediction of the emotions are based on RPM, so accurate RPM estimation is very critical. For this purpose, the raw respiration data are processed to clean the noise. Figure 9a,b shows the noisy and clean data, respectively. It can be seen that the processed data provides smooth peaks as compared to the noisy data and peak estimation is easy in the cleaned data. The cleaned data are used to detect and count the peaks using the defined α threshold, as shown in Figure 10. RPM is counted using Equation (3).

**Figure 11:**
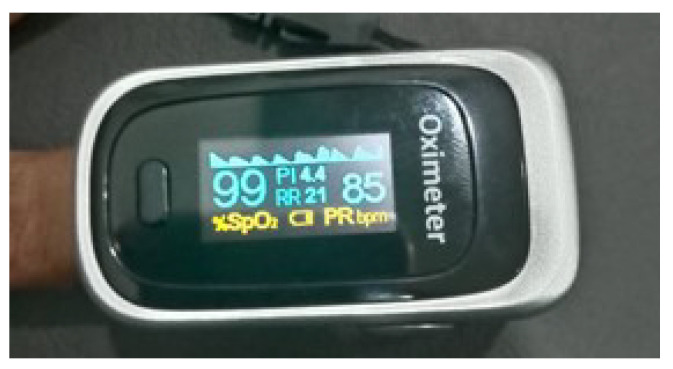
Pulse oximeter used for measuring RPM.

For validating the performance and extent of accuracy for RPM, experiments are performed using different participants. The procedure was replicated several times against various participants. The obtained RPM is validated against a commercial Pulse oximeter used in [44] and shown in Figure 11.

RPM validation experiments are performed for both static and dynamic environments where the subject sits on a chair in the static movement while the subject’s measurements are taken for the dynamic environment when reading a book. Ten people are chosen for the validation experiment including five males and five females between the age of 25 to 30 years. The person operating radar signals informs the subjects and triggers the radar and in the meantime, the subject triggers the wearing pulse oximeter. The hand movement of the subject while he triggers the pulse oximeter does not affect the RPM obtained from the chest movement signal recorded by the UWB radar. Individuals are told to sit comfortably in a chair facing the radar, with the pulse oximeter attached to their left index finger. Each subject’s chest movements are recorded twice for 1 min each time. RPM using IR-UWB data is calculated following the above-described procedure.

Table 2 displays the results of calculated RPM and commercially available pulse oximeter. Results show that the RPM calculated by the proposed method is in complete agreement with the pulse oximeter results validating the accuracy of the proposed method.

To ensure that the movement of participants does not affect the performance of the proposed system, experiments are carried out while subjects are reading a book. A pulse oximeter is attached to the left index finger, and radar is placed in front of the subject. The experiment included eight male individuals, and data was collected using a pulse oximeter and a radar at the same time. Experimental results for the dynamic environment are provided in Table 3. Results suggest that the method is robust and can perform well even in dynamic environments with slight variation in the measured RPM. For example, for participants 1, 2, 4, and 5 a difference of 1 RPM is found between the pulse oximeter RPM and the calculated RPM, however, it is not significant to influence the emotion detection process.

For corroborating the hypothesis that there is no statistically significant difference between the pulse oximeter and calculated RPMs, a T-test is performed. Table 4 shows the value of the statistical T-test to evaluate the RPM for static and dynamic movement of the participant. Similarly, the RPM statistics of males and females are also analyzed. In both cases, the RPM data is statically equal indicating that there is no significant difference in static body RPM and dynamic body RPM and there is also no difference between female and male RPMs.

### 5.2. Results of RPM for Male and Female Participants

To analyze the difference of RPM in different gender, a separate set of experiments has been carried out. Such experiments are performed with a two-fold purpose. First, the difference in male and female participants can be analyzed using the obtained RPM. Second, the RPM difference is investigated for each gender with respect to the three emotions studied in this study. The average RPM of males and females when different emotions are induced is presented in Table 5. Two important findings are obtained during experiments. First, there is an observable difference in RPM for males and females. Second, the RPM of males and females is affected differently when faced with different emotions. For happiness, male participants show a higher RPM indicating that they are aroused highly as compared to female participants. On the other hand, fear emotion is more experienced by female participants. Regarding the disgust emotion, the RPM is almost similar for both males and females. We performed statistical analysis on female and male RPM using statistical *t*-test and results of *t*-test show that male and female RPMs are not statistically different regarding each emotion. Both RPMs show the same statistics as *t*-test findings given in Table 4 indicates.

### 5.3. Results for Emotion Recognition

For capturing the data during different induced emotions, videos are played on the laptop remotely controlled using the team-viewer. The Chest movement of each subject is acquired three times by showing three videos of each emotion for three consecutive days (one emotion per day). The RPM is estimated from the chest movement of subjects using the proposed approach. All classifiers are trained and evaluated to compare their performance and select the best fit model. A structured dataset is maintained comprising of RPM, gender (’0’ for ’female’ and ’1’ for ’male’), and age of the subject along with the labels ’0’ for ’happy’, ’1’ for ’disgust’ and ’2’ for ’fear’. A few sample records from the dataset are shown in Table 6.

**Figure 12:**
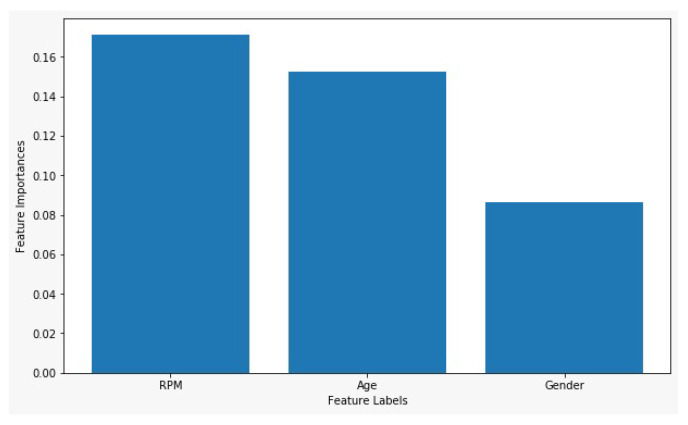
Feature importance of dataset attributes using RF.

The use of the RPM feature is validated through feature importance. Figure 12 shows the feature importance using the random forest (RF) algorithm. It shows that RPM has significant importance for emotion classification as compared to age and gender. While gender feature score is the lowest which also indicates that the female and male do not impact the classification accuracy.

The dataset contains 315 tuples in total with 105 for each emotion. The dataset is divided into two set for training and testing in different ratios. The ML models are fed with a non-standardized feature vector including gender, RPM (average respiration rate of 5 m), age, and emotion as input. Following that classifiers are evaluated using previously split test data. Table 7 shows the classification results using 90:10 train-test split. GBM obtains the highest accuracy of 0.79 following by HV and SV with 0.77 and 0.74, respectively.

Reducing the train data degrades models’ performance as expected. The highest accuracy is now 0.78 by GBM, as shown in Table 8. HV obtains the 2nd highest accuracy with 0.75 followed by 0.73 each from SV and XGB. Despite the reported accuracy scores, the F1 score is preferred over accuracy as it incorporates both precision and recall and has been regarded as a more balanced measure to evaluate the performance of a model. The proposed ensemble SV obtains the highest F1 score of 0.76. Similarly, XGB shows an F1 score of 0.74 which is also significant considering that the problem is a multiclass classification.

The performance of the models is further reduced once the ratio of the train-test split is changed to 70:30, as shown in Table 9. Although the performance is degraded in terms of accuracy, yet, the F1 score is improved on average with six out of seven classifiers having 0.70 plus F1 score which is not the case for 90:10 or 80:20 train-test split.

For further analysis of the models’ performance, experiments are performed using 60:40 and 50:50 train-test splits and results are shown in Table 10 and Table 11, respectively. Results indicate that both the accuracy, as well as, F1 score has been impacted on account of the decrease in the training data. One important point to be mentioned is the consistent performance of GBM and SV which shows better performance for all train-test splits and be among the significantly performing models.

The performance of all models is more significant with 70:30 and 80:20 train-test split ratios, especially, in terms of F1-Score. With enough data for models’ training, the results are significantly better. The used dataset is multiclass and also small in size, so considering the model’s overfitting problem F1 Score can be more appropriate for a fair comparison. That is the reason for selecting the F1 score as the primary performance evaluation measure. Results indicate that the proposed SV is significant with the highest F1 score of 0.76 with an 80:20 splitting ratio. The significant performance of SV is because of probability-based ensemble architecture. Other tree-based models, such as ETC, GBM, ADAB, and XGB also show good results.

### 5.4. Results of K-Fold Cross Validation

For validating the performance of machine learning models, 10-fold cross-validation has been performed and the results are given in Table 12. The proposed SV achieves the highest accuracy of 0.66 with a ±0.33 standard deviation (SD). Proposed HV and XGB are just behind the ESV with 0.65, and 0.64 accuracy scores and ±0.29, ±−0.40 SD, respectively.

### 5.5. Experimental Results Using Deep Learning Models

Results of deep learning models are shown in Table 13. Results indicate that the performance of deep learning models is not significant in comparison to machine learning models. CNN obtains the best F1 score of 0.54 with an 80:20 ratio while MLP achieves a 0.61 F1 score with a 50:50 ratio. On average, MLP shows better performance as compared to the CNN model. The maximum accuracy of 0.72 is obtained by MLP. Primarily, deep learning models tend to show better performance when fed with a large amount of training data. For the current study, the small amount of the collection may be a probable reason for the poor performance of the deep learning models. CNN required a large feature set for better training and in this study dataset features set is small which leads to a poor fit of the CNN model. While MLP is somehow better in comparison with CNN because of MLP’s simple architecture as compared to CNN. MLP is a basic model which can be good even on a small dataset because of its simple architecture.

### 5.6. Comparison with Recent Research Studies

The performance of the proposed approach is compared to other research works that utilize respiration rates for emotion classification. Table 14 indicates that [35,38] shows slightly better accuracy than the proposed system. However, ref. [35] uses both ECG and respiration data for emotion classification, and data from multiple sensors tend to show better results. Electrodes were used to record ECG signals while respiration rate was calculated from the inhale and exhale of gases through the oxygen mask subject wore during the data collection. In contrast, the proposed approach obtains very similar results using only the respiration rate. A Biopac device and mouthpiece were used by [38] to collect respiration rate and respiration patterns and the proposed approach is an invasive method, contrary to the non-invasive approach presented in this study. The devices used by [35,38] for data collection need to be worn on the body that requires correct placement and makes the subject responsive and overly reacting resulting in inaccurate data. The proposed approach obtains respiration rates from chest movements using UWB radar. UWB radar is a non-invasive technology that can detect chest movements across a range of 0.2 to 1.6 m. Other movements such as blinking, head movements, and so on, can be identified and filtered.

## 6. Conclusions and Future Work

Emotions are conscious and/or unconscious mental reactions from humans to various events, objects, and situations and are part and parcel of human life. Although emotion recognition is important for humans to respond appropriately, the past few years are marked with increased research in emotion recognition for human therapy, the latest human-computer interface, and advanced humanoids. Several kinds of physiological signals can be utilized for the task at hand with EEG, ECG, GSR, and respiration-based methods as the most famous. Predominantly, such methods involve placing devices on the head and chest or attaching sensors to various limbs. Consequently, such methods introduce inconvenience for humans which leads to noisy and erroneous data and wrong emotion recognition.

This study presents a novel non-invasive emotion recognition approach based on human respiration patterns. The respiration data are obtained using the novel use of IR-UWB radar for three emotions including happiness, fear, and disgust. These emotions are induced using movie clips while the chest movements of thirty-five participants are collected including both males and females. RPM calculated by the proposed method is validated by a commercial Pulse Oximeter which shows a 100% accuracy. RPM and other features are fed into the machine and deep learning models for training and testing. Additionally, hard and soft voting-based ensemble models are proposed for emotion recognition as well. Extensive experiments have been carried out involving different train-test splits. Results indicate that using the proposed novel non-invasive approach, a 76% F1 score can be obtained from the proposed SV with an 80:20 train-test split. Besides the basic emotion recognition, separate experiments are performed based on gender which provides insight on gender-based behavior during different emotions. Male participants show high arousal in happiness while fear emotion is more prevalent and intense in female participants. The emotion intensity for disgust is almost similar for both males and females. This study lays the foundation for IR-UWB based non-invasive emotion recognition, yet, only three emotions are studied at the moment. In the future, the dataset will be extended to add further emotions, as well as, improve the accuracy of machine learning, and deep learning models by incorporating additional features.

## Figures and Tables

**Table 1:** List of hyperparameters used for experiments.

Classifier	Hyperparameters
ETC	n_estimators = 200, random_state = 100, max_depth = 200, min_samples_split = 80
ADB	n_estimators = 50, random_state = 100, learning_rate = 1.0
GBM	max_depth = 100, n_estimators = 100, random_state = 42, min_samples_split = 90, min_samples_leaf = 10
KNN	n_neighbors = 7, leaf_size = 1
XGB	n_estimators = 50, max_depth = 100, learning_rate = 0.8
HV	Base learners = XGB, ADA, GBM, KNN, voting = hard/majority
SV	Base learners = XGB, ADA, GBM, KNN, voting = soft

**Table 2:** Results for validation experiments for RPM.

Subject	Respiration Rate	
	Pulse Oximeter	Proposed Method
Participant 1	16	16
Participant 1	19	19
Participant 2	21	21
Participant 2	22	22
Participant 3	15	15
Participant 3	12	12
Participant 4	15	15
Participant 4	17	17
Participant 5	18	19
Participant 5	16	16
Participant 6	18	18
Participant 6	20	20
Participant 7	17	17
Participant 7	19	19
Participant 8	17	17
Participant 8	15	15
Participant 9	12	12
Participant 9	14	14
Participant 10	18	18
Participant 10	17	17

**Table 3:** Results for validation experiments for RPM in dynamic environment.

Subject	Respiration Rate	
	Pulse Oximeter	Proposed Method
Participant 1	15	14
Participant 1	17	16
Participant 2	16	16
Participant 2	14	15
Participant 3	17	17
Participant 3	20	19
Participant 4	16	16
Participant 4	18	17
Participant 5	14	15
Participant 5	20	19
Participant 6	17	18
Participant 6	16	17
Participant 7	18	17
Participant 7	17	19
Participant 8	16	17
Participant 8	14	14

**Table 4:** Statistical T test to validate the RPM results.

Statistical T Test	Static/Dynamic	Male/Female
df	30	30
cv	1.697	1.697
*p*-value	0.920	0.945
t-statistic	0.920	0.069
alpha	0.05	0.05

**Table 5:** Average RPM of males and females During different emotions.

Gender	Average RPM		
	Happiness	Disgust	Fear
Male	19.56	19.35	19.85
Female	18.47	19.38	20.54

**Table 6:** Sample records from the collected dataset.

Subject	RPM	Gender	Age	Emotion
Participant 1	20	1	26	0
Participant 2	20	1	27	0
Participant 3	21	0	27	0
Participant 4	22	1	24	1
Participant 5	23	0	26	1
Participant 6	20	1	30	1
.	.	.	.	.
.	.	.	.	.
.	.	.	.	.

**Table 7:** Performance metrics with 90:10 train and test size.

Classifier	Accuracy	Precision	Recall	F1 Score
ETC	0.70	0.67	0.68	0.66
ADB	0.70	0.69	0.70	0.69
GBM	0.79	0.69	0.70	0.69
KNN	0.73	0.59	0.61	0.59
XGB	0.69	0.69	0.69	0.69
EHV	0.77	0.66	0.66	0.66
ESV	0.74	0.72	0.73	0.72

**Table 8:** Performance metrics with 80:20 train and test size.

Classifier	Accuracy	Precision	Recall	F1 Score
ETC	0.68	0.68	0.68	0.68
ADB	0.68	0.68	0.71	0.68
GBM	0.78	0.71	0.73	0.71
KNN	0.72	0.68	0.68	0.68
XGB	0.73	0.75	0.73	0.74
EHV	0.75	0.71	0.73	0.71
ESV	0.73	0.76	0.77	0.76

**Table 9:** Performance metrics with 70:30 train and test size.

Classifier	Accuracy	Precision	Recall	F1 Score
ETC	0.67	0.67	0.69	0.68
ADB	0.70	0.72	0.73	0.72
GBM	0.78	0.72	0.72	0.72
KNN	0.70	0.72	0.71	0.72
XGB	0.72	0.72	0.72	0.72
EHV	0.73	0.73	0.73	0.73
ESV	0.74	0.74	0.74	0.74

**Table 10:** Performance metrics with 60:40 train and test size.

Classifier	Accuracy	Precision	Recall	F1 Score
ETC	0.67	0.67	0.71	0.67
ADB	0.68	0.68	0.70	0.68
GBM	0.78	0.73	0.74	0.73
KNN	0.71	0.63	0.63	0.63
XGB	0.68	0.69	0.68	0.68
EHV	0.71	0.71	0.74	0.71
ESV	0.75	0.71	0.71	0.71

**Table 11:** Performance Metrices with 50:50 train and test size.

Classifier	Accuracy	Precision	Recall	F1 Score
ETC	0.63	0.65	0.63	0.63
ADB	0.68	0.68	0.68	0.67
GBM	0.70	0.70	0.70	0.70
KNN	0.53	0.55	0.53	0.52
XGB	0.67	0.67	0.67	0.67
EHV	0.72	0.62	0.62	0.61
ESV	0.72	0.71	0.72	0.71

**Table 12:** Performance of machine learning models with 10-fold cross validation.

Classifier	Accuracy (±Std. Dev.)
ETC	0.64 (±0.35)
ADB	0.61 (±0.37)
GBM	0.65 (±0.36)
KNN	0.57 (±0.22)
XGB	0.64 (±0.40)
EHV	0.65 (±0.29)
ESV	0.66 (±0.33)

**Table 13:** Performance of deep learning models with different train-test split ratios.

Ratio	Classifier	Accuracy	Precision	Recall	F1 Score
90:10	MLP	0.62	0.65	0.62	0.60
	CNN	0.40	0.51	0.40	0.42
80:20	MLP	0.67	0.67	0.67	0.66
	CNN	0.55	0.62	0.55	0.54
70:30	MLP	0.62	0.62	0.62	0.61
	CNN	0.40	0.41	0.40	0.40
60:40	MLP	0.63	0.64	0.63	0.61
	CNN	0.38	0.53	0.38	0.40
50:50	MLP	0.72	0.62	0.62	0.61
	CNN	0.40	0.51	0.40	0.42

**Table 14:** Performance comparison with different emotion detection approaches.

Reference	Feature Vector	Accuracy
[33]	Respiration rate interval, low frequency, heart rate, high frequency, RSA power, RSA frequency, and RSA amplitude breathing frequency, breathing amplitude, RSA amplitude, ratio to respiratory oscillation, respiratory and RSA frequency difference, the phase difference of respiration and RSA, the slope of phase difference, and standard deviation.	73% for liking, 72% for arousal, and 70% for valence
[35]	Root-mean-square, intrinsic mode functions, ‘mean’, ‘max’, from respiration and ECG signal Respiration rate and heart rate	80%
[36]	Heart rate variability from ECG signal, respiration rate and amplitude from respiration signal.	68.5% for arousal, 68.75% for valence
[37]	Statistical features average, maximum, minimum, and standard deviation etc.	55.45% for arousal, 59% for valence
[38]	Air flow rate and volume	80%
[39]	Different statistical and time domain features	79.2% for relax vs. joy, 77.8% for joy vs. sad, and 77.3% for joy vs. anger
Current study	RPM, Age and, Gender	79%

## Data Availability

Not Applicable.
